# Netrin-1 Reduces Monocyte and Macrophage Chemotaxis towards the Complement Component C5a

**DOI:** 10.1371/journal.pone.0160685

**Published:** 2016-08-10

**Authors:** Lewis Taylor, Maximillian Hugo Brodermann, David McCaffary, Asif Jilani Iqbal, David R. Greaves

**Affiliations:** Sir William Dunn School of Pathology, University of Oxford, Oxford, United Kingdom; University of São Paulo FMRP/USP, BRAZIL

## Abstract

Netrin-1, acting at its cognate receptor UNC5b, has been previously demonstrated to inhibit CC chemokine-induced immune cell migration. In line with this, we found that netrin-1 was able to inhibit CCL2-induced migration of bone marrow derived macrophages (BMDMs). However, whether netrin-1 is capable of inhibiting chemotaxis to a broader range of chemoattractants remains largely unexplored. As our initial experiments demonstrated that RAW264.7 and BMDMs expressed high levels of C5a receptor 1 (C5aR1) on their surface, we aimed to determine the effect of netrin-1 exposure on monocyte/macrophage cell migration induced by C5a, a complement peptide that plays a major role in multiple inflammatory pathologies. Treatment of RAW264.7 macrophages, BMDMs and human monocytes with netrin-1 inhibited their chemotaxis towards C5a, as measured using two different real-time methods. This inhibitory effect was found to be dependent on netrin-1 receptor signalling, as an UNC5b blocking antibody was able to reverse netrin-1 inhibition of C5a induced BMDM migration. Treatment of BMDMs with netrin-1 had no effect on C5aR1 proximal signalling events, as surface C5aR1 expression, internalisation and intracellular Ca^2+^ release following C5aR1 ligation remained unaffected after netrin-1 exposure. We next examined receptor distal events that occur following C5aR1 activation, but found that netrin-1 was unable to inhibit C5a induced phosphorylation of ERK1/2, Akt and p38, pathways important for cellular migration. Furthermore, netrin-1 treatment had no effect on BMDM cytoskeletal rearrangement following C5a stimulation as determined by microscopy and real-time electrical impedance sensing. Taken together these data highlight that netrin-1 inhibits monocyte and macrophage cell migration, but that the mechanism behind this effect remains unresolved. Nevertheless, netrin-1 and its cognate receptors warrant further investigation as they may represent a potential avenue for the development of novel anti-inflammatory therapeutics.

## Introduction

Inflammation is a coordinated host response to local injurious stimuli [1]. It triggers a protective tissue reaction to dilute, isolate, and destroy the causative agent and initiate repair. Macrophages are fundamental to this process as cellular mediators of both acute and chronic inflammation. Their directed migration to and from sites of inflammation is controlled by a range of chemoattractant mediators including leukotrienes, secreted chemokines, and complement peptides [2–4]. With ever more sophisticated methods to study cell migration, an increasing number of molecules are being shown to modulate macrophage chemotaxis, one example being the cellular guidance signal netrin-1 [5].

Netrin-1’s classical role is in the development of the central nervous system (CNS). It is secreted at the ventral midline of the embryonic neural tube and creates a gradient along which neurons differentially migrate [6]. Axonal chemoattraction is mediated by the netrin-1 receptors DCC and neogenin [7], whereas UNC5b broadly facilitates axonal chemorepulsion [8]. More recently netrin-1 has been shown to influence cell migration beyond the CNS, most notably as an immunomodulatory protein in the setting of inflammation [5, 9].

Several published studies have shown the wide-ranging actions of netrin-1 across the immune system. These include the observations that netrin-1 dampens inflammatory peritonitis in vivo [10] and that netrin-1 expression in the adipose tissue of obese individuals favours macrophage retention, characteristic of chronic inflammation and insulin resistance associated with type 2 diabetes [11]. Most important to our investigation was the observation made by van Gils *et al* that netrin-1, secreted by macrophages within atherosclerotic lesions, acts to promote atherosclerosis by inhibiting macrophage emigration from plaques in an UNC5b dependent manner [12]. In particular, it was shown that netrin-1 reduced macrophage chemotaxis *in vitro* towards the chemokines CCL2 and CCL19, which are respectively implicated in monocyte recruitment to, and macrophage egress from atherosclerotic plaques [12–14]. Indeed, we were able to replicate and extend this previous finding, as we demonstrated that netrin-1 was able to inhibit BMDM migration towards CCL2.

Although CC chemokines play a key role throughout an inflammatory response, other chemoattractants are equally important for monocyte recruitment and macrophage activation. However, the action of netrin-1 on non-CC chemokine mediated monocyte and macrophage chemotaxis is relatively poorly studied. In contrast to CC chemokine receptors, we observed that RAW264.7 and bone marrow derived macrophages (BMDMs) expressed high surface levels of C5a receptor 1 (C5aR1). C5a is a complement peptide that has an important role in innate immunity with potent chemotactic and anaphylatoxic properties that upregulate endothelial adhesion molecules, increase vascular permeability, and localise leukocytes and inflammatory molecules at sites of infection [15]. As such, C5a is directly implicated in several inflammatory pathologies including glomerular disease, ischaemia reperfusion injury, and degenerative diseases such as macular degeneration [16]. In the context of cardiovascular disease, netrin-1 is already being considered as a therapeutic agent [17] with human netrin-1 gene delivery resulting in significantly reduced atherosclerotic plaque formation in low-density lipoprotein receptor knockout mice being fed a high cholesterol diet [18]. We therefore aimed to explore whether netrin-1 would limit C5a induced macrophage chemotaxis as it does with macrophage chemotaxis towards CCL2 and CCL19.

Using two different real-time chemotaxis platforms, we show for the first time that netrin-1 inhibits human monocyte and murine macrophage migration towards C5a. We explored the possible mechanism behind the reduction both at C5aR1 proximal events and at signalling events further downstream of receptor ligation. We conclude that netrin-1 inhibition of macrophage chemotaxis towards C5a is not mediated through C5a receptor internalisation or changes in macrophage Ca^2+^ release, cell spreading, or altered PI3K/MAPK signalling. Although the mechanism remains unresolved, a better understanding of netrin-1’s impact on macrophage chemotaxis could identify further therapeutic avenues to explore in the development of novel anti-inflammatory drugs.

## Materials and Methods

### Reagents

Murine CCL2, C5a, netrin-1 and the rat UNC5b blocking antibody were obtained from R & D systems (Abingdon, UK). CIM-16 plates were purchased from Cambridge Bioscience (Cambridge, UK). complete, EDTA free protease inhibitor cocktail tablets were purchased from Roche (Burgess Hill, UK). Rabbit anti-phospho-ERK1/2 (D13.14.4E), rabbit anti-phospho-Akt (D9E), rabbit anti-phospho-p38 (D3F9), rabbit total ERK1/2 (137F5) and rabbit total p38 (D13E1) were purchased from Cell Signalling Technologies (Danvers, MA, USA). HRP-conjugated Goat anti-rabbit secondary and Bio-gel polyacrylamide beads (P-100 fine 45–90 μm) were purchased from Biorad (Hertfordshire, UK). Matrigel and all cell culture flasks and vessels were purchased from Corning (Flintshire, UK). Fura-2 AM and Alexa Fluor® 488 phalloidin were purchased from Life Technologies. Phosphatase inhibitor cocktail 2, cell culture medium and all other laboratory chemicals were purchased from Sigma Aldrich (Dorset, UK).

### Animals

C57BL/6J mice were obtained from Harlan Laboratories (Oxfordshire, UK). All animal studies were conducted with ethical approval from the Dunn School of Pathology Local Ethical Review Committee and in accordance with the UK Home Office regulations (Guidance on the Operation of Animals (Scientific Procedures) Act 1986). Animals were humanely killed by exposure to carbon dioxide gas in a rising concentration, as set out in Schedule one of the Animals (Scientific Procedures) Act 1986, and all efforts were made to minimise distress and suffering of the animals used in this study.

### Generation of bone marrow derived macrophages

Bone marrow cells from the tibiae and femur of male C57BL/6J mice were collected, counted, and 4x10^6^ cells seeded into 10 cm non-tissue culture treated petri dishes in 10 ml of differentiation medium (DMEM supplemented with 10% fetal calf serum (FCS), 15% L929 cell conditioned supernatant, 2 mM l-glutamine and 100 U/ml penicillin/streptomycin). Three days later, an additional 5 ml of differentiation medium was added. BMDMs were then collected after 7 days by the addition of phosphate buffered saline (PBS) containing 2 mM EDTA and gentle agitation. BMDMs were then resuspended in chemotaxis buffer (RPMI 1640 supplemented with 0.5% BSA and 25 mM HEPES), counted and adjusted to the desired cell concentration.

### Cell culture

The RAW264.7 murine macrophage cell line [19], originally from the ATCC, was a kind gift from Professor Siamon Gordon and was cultured using DMEM supplemented with 10% FCS and 100 U/ml penicillin/streptomycin. RAW264.7 macrophages were seeded into T75 non-tissue culture treated flasks (2x10^6^ cells/flask) and maintained at 37°C, 5% CO_2_. When approximately 80–90% confluent, the medium was removed and the cells lifted from the flask by the addition of PBS containing 10 mM EDTA followed by firm tapping. Cells were then resuspended in PBS and counted. For experimental usage, cells were then pelleted and resuspended in chemotaxis buffer to give the desired cell concentration. For propagation of the cell line, cells were resuspended in DMEM supplemented with 10% FCS and 100 U/ml penicillin/streptomycin and seeded into T75 flasks as detailed above. For all experiments, RAW264.7 macrophages were used at a passage number of 10 or lower.

### Bio-gel elicitation of peritoneal exudate cells and bio-gel macrophage enrichment

Male C57BL/6J mice were injected intraperitoneally with 1 ml of sterile 2% bio-gel polyacrylamide beads (P100 fine, 45–90 μM) suspended in PBS. After four days, mice were sacrificed and the elicited cells collected by peritoneal lavage with 10 ml ice cold PBS containing 2 mM EDTA. Subsequently, peritoneal bio-gel elicited cells were pelleted by centrifugation at 200 xg for 5 min at 4°C and then re-suspended in 10 ml chemotaxis buffer before being transferred into 10 cm petri dishes (one per mouse; non-tissue culture treated) and left for 2 hours at 37°C, 5% CO_2_. The dishes were then washed three times with 10 ml PBS to remove any non-adherent cells and the medium replaced with 10 ml chemotaxis buffer. After being left overnight at 37°C, 5% CO_2_, adherent macrophages were collected by the addition of PBS containing 10 mM EDTA and gentle agitation. Macrophages were then pelleted by centrifugation at 200 xg for 5 min at 4°C, resuspended in chemotaxis buffer, counted, and then adjusted to the desired cell concentration. These cells are subsequently referred to as bio-gel elicited macrophages and we have previously demonstrated that these are highly enriched for macrophages [20].

### Isolation of human monocytes

Leukocyte cones were obtained from the NHS blood transfusion service or peripheral blood was taken from healthy volunteers, with informed consent and appropriate local ethics approval, and peripheral blood mononuclear cells (PBMCs) were obtained by Ficoll gradient centrifugation. Leukocyte cones were diluted 1 in 2 with PBS, whereas fresh blood was not, and 15 ml then layered on top of 5 ml Ficoll-Paque Plus (GE Healthcare) before centrifugation at 900 xg for 20 min at RT. The PBMC layer was carefully harvested and then washed repeatedly using 50 ml PBS and centrifugation at 300 xg for 5 min until the supernatant became clear. CD14^+^ monocytes were then isolated from the PBMCs by positive magnetic separation using human anti-CD14 coupled microbeads following the manufacturer’s instructions (Miltenyi Biotec). CD14^+^ monocytes were frozen for long term storage in FCS supplemented with 10% DMSO.

### ACEA xCELLigence real-time chemotaxis assay

Real-time chemotaxis analysis was conducted as previously described [2]. Briefly, vehicle (chemotaxis buffer alone), CCL2 (10 nM) or C5a (1.25, 2.5, 5 or 10 nM) was added to the lower chamber of a CIM-16 plate (160 μl/well). The upper chamber was subsequently attached and 50 μl of pre-warmed chemotaxis buffer added to each of the upper chambers. Following equilibration at RT for 30 min, the plate was transferred into the RTCA-DP system for background analysis. Simultaneously, RAW264.7 macrophages, BMDMs or human monocytes (1x10^5^ cells/well) were treated with either vehicle or 3 nM netrin-1 for 45 min at 37°C, 5% CO_2_. For experiments with bio-gel elicited macrophages 4x10^5^ cells/well were used. Cells were then added to all upper wells (50 μl/well) and plate replaced into the RTCA-DP system. Cell Index (CI) measurements were then taken every 5 seconds over the 3–4 hour assay period. Change in Cell Index (Δ Cell Index) was calculated as maximum cell index minus minimum cell index and pooled data are displayed as a fold change relative to cells migrating towards vehicle alone. For the heat inactivation experiments, netrin-1 was incubated at 75°C for 25 min prior to BMDM treatment. For the UNC5b receptor blocking experiments, BMDMs were co-incubated with netrin-1 (3 nM) and an UNC5b blocking antibody (10 μg/ml) for 45 min at 37°C, 5% CO_2_, prior to being placed into the upper chamber of a CIM-16 chemotaxis plate.

### ACEA 96 well ECIS cell spreading assay

Chemotaxis buffer (50 μl) was added to the wells of a 96 well E-plate and a background measurement taken. Afterwards, BMDMs were added to each well (50 μl—20,000 cells/well) and left for 2–3 hours at 37°C, 5% CO_2_ to adhere to the bottom gold electrode, with CI measurements made every 15 min. Cells were then treated with vehicle (chemotaxis buffer) or 3 nM netrin-1 for 45 min prior to being stimulated with vehicle or 10 nM C5a. CI measurements were taken every 5 s for 1 hour after agonist addition. Data are displayed as change in CI from the point of agonist addition (Δ Cell Index) and response was calculated as maximum CI–CI at point of agonist addition.

### IncuCyte® ZOOM real-time chemotaxis assay

BMDMs or RAW264.7 macrophages were incubated with either vehicle (RPMI + 10% FCS), 3 nM or 30 nM netrin-1 for 45 min at 37°C, 5% CO_2_ prior to being placed into the upper chamber of an IncuCyte® ClearView chemotaxis plate (60 μl—5000 cells/well), which had been coated with Matrigel (50 μg/ml for 30 min at 37°C and 30 min at RT). The plates were then left for 1 hour at RT to allow cell adhesion to the topside of the chemotaxis well. Vehicle or 100 nM C5a (200 μl) was added to the lower chamber of a ClearView chemotaxis plate and the top section carefully placed into the bottom. The plate was then placed into the IncuCyte® ZOOM microscope, which was housed inside a humidified incubator set to 37°C. The microscope was then set to take images of the top and underside of each well every 2 hours. Migration was then quantified by the IncuCyte® analysis software as the appearance of in focus cells, and the respective surface area they occupy (defined as total phase object area in μm^2^/well), on the underside of the well. To validate equal cell loading, the surface area occupied by cells on the topside of the well at time point zero was calculated.

### IncuCyte® ZOOM cell spreading assay

BMDMs were seeded into 96 well black walled plates (5,000 cells/well) in chemotaxis buffer and allowed to adhere for 2 hours at 37°C, 5% CO_2_. Cells were then treated with either vehicle (chemotaxis buffer) or 3 nM netrin-1 for 45 min at 37°C, 5% CO_2_ prior to being stimulated with either vehicle or 10 nM C5a for 5 or 30 min. Following stimulation, cells were fixed by the addition of 4% formaldehyde for 15 min at RT. Cells were washed twice with PBS and 0.5% triton X-100 was added for 5 min to permeabilise the cell membrane. The cells were then washed twice with PBS and 5 U/ml Alexa Fluor® 488 phalloidin was added to each well for 20 min at RT to visualise F-actin. Cells were then washed twice with PBS and the plate placed into the IncuCyte® ZOOM microscope and four images per well were then taken by the microscope. The IncuCyte® analysis software then determined individual cell areas by recognising unique green fluorescent objects in each image, and then calculated the average cell area for each well.

### Cell viability assay

BMDMs were seeded into 96 well black walled plates (50,000 cells/well) and allowed to adhere for 2 hours at 37°C, 5% CO_2_. Cells were then treated with vehicle, 3 nM or 30 nM Netrin-1 for 45 min, 4 hours or 24 hours. Afterwards, cell viability was determined using the CellTiter-Glo^®^ luminescent cell viability assay (Promega, Southampton), which determines cell viability by quantification of ATP, following the manufacturer’s protocol. Briefly, an equal volume of CellTiter-Glo^®^ reagent was added to each well and the plates were left for 15 min before luminescence was quantified using a PHERAstar plate reader (BMG Labtech, Aylesbury, UK).

### Flow cytometry

Cells (5 x 10^5^) were placed into polypropylene tubes and pelleted by centrifugation at 200 xg for 5 min at 4°C. These were then resuspended in FACS buffer (PBS containing 2% FCS, 25 mM HEPES and 5 mM EDTA) supplemented with mouse IgG and mouse SeroBlock FcR^®^ (AbD Serotec, Oxford, UK) and left on ice for 30 min. Specific staining was conducted using the following antibodies and appropriate isotype controls: F4/80 (1:100, FITC, clone CI:A3-1, Abcam, Cambridge, UK), Ly-6B.2 (1:100, Alexa Fluor® 647, clone 7/4, Abd Serotec), CCR2 (1:100, PE, clone 475301, R & D systems) and C5aR1 (1:100, PE, clone 20/70, Biolegend) for 1 hour on ice. Cells were then pelleted by centrifugation at 200 xg for 5 min at 4°C and resuspended in 1% formaldehyde. For C5aR1 internalisation experiments, cells were treated with vehicle or 3 nM netrin-1 for 45 min at 37°C, 5% CO_2_ prior to being stimulated with 10 nM C5a for either 5 or 30 min. C5aR1 surface staining was then conducted as described above. For measurement of intracellular receptor levels, cells were first fixed in 1% formaldehyde for 15 min. Next, they were resuspended in 0.5% saponin for 15 min at RT to permeabilise the cell membrane. Cells were then incubated with fluorescently conjugated receptor antibodies in 0.5% saponin for 1 hour on ice, washed with FACS buffer and then resuspended in 1% formaldehyde. Analysis was conducted using a Dako Cyan ADP flow cytometer (Beckman Coulter Ltd, High Wycombe, UK) and FlowJo software (V10, Tree Star, Ashland, USA).

### Intracellular cAMP assay

Intracellular cAMP levels were measured using Discoverx cAMP Hunter™ eXpress kits (Discoverx, Birmingham, UK) following the manufacturer’s protocol. Briefly, CHO-K1 cells stably expressing human C5aR1 receptor were plated into ½ area 96 well plates (15,000 cells/well) and incubated at 37°C, 5% CO_2_ for 24 hours. Cells were then treated with either vehicle (Cell assay reagent) or 3 nM netrin-1 for 45 min at 37°C, 5% CO_2_ before being stimulated with either vehicle or C5a at the indicated concentration for 30 min at 37°C, 5% CO_2_. Cell lysis and cAMP detection were then performed as per the manufacturer’s protocol. Luminescence measurements were taken using a PHERAstar microplate reader.

### C5aR1 β-arrestin recruitment assay

Recruitment of β-Arrestin to C5aR1 was measured using the Discoverx PathHunter® eXpress β-Arrestin GPCR Assay following the manufacturer’s protocol. Briefly, CHO-K1 cells stably expressing murine C5aR1 were seeded into ½ area 96 well plates (15,000 cells/well) and incubated at 37°C, 5% CO_2_ for 48 hours. Cells were then treated with either vehicle (Cell assay reagent) or 3 nM netrin-1 for 45 min at 37°C, 5% CO_2_ prior to stimulation with vehicle or C5a at the indicated concentration for 90 min at 37°C, 5% CO_2_. Cell lysis and detection of total recruited β-arrestin was determined following the manufacturers protocol. Luminescence measurements were taken using a PHERAstar microplate reader.

### Intracellular Ca^2+^ measurement

BMDMs were seeded into black walled 96 well plates (50,000 cells/well) and left for 2 hours at 37°C, 5% CO_2_ to adhere. The medium was then removed and replaced with RPMI containing 4 μM Fura 2-AM (Life Technologies) supplemented with either vehicle or 3 nM netrin-1. Plates were then left for 45 min at RT in the dark. Afterwards, cells were washed twice with PBS, the medium replaced with Fluorobrite™ DMEM (Life technologies) and the plate placed into a PHERAstar microplate reader set to 37°C. BMDMs were then stimulated with either vehicle or 10 nM C5a and changes in Fura-2 fluorescence were measured using excitation wavelengths of 340 nm and 380 nm and an emission wavelength of 510 nm. The change in the ratio of 340/380 nm stimulated emission at 510 nm was then calculated (referred to as Δ340/380 nm).

### Western blotting

BMDMs were seeded into tissue culture treated 6 well dishes (2x10^6^ cells/well) in chemotaxis buffer and allowed to adhere for 2 hours at 37°C, 5% CO_2_. The cells were then treated with vehicle (chemotaxis buffer) or 3 nM netrin-1 for 45 min at 37°C, 5% CO_2_ prior to stimulation with vehicle or 10 nM C5a for 5 or 30 min. After stimulation, the medium was rapidly removed and the 6 well dishes placed at -80°C. Cell lysates were prepared by the addition of cell lysis buffer (150 mM NaCl, 0.8 mM MgCl_2_, 5 mM EGTA, 50 mM HEPES, 1% IGEPAL CA-630) supplemented with protease and phosphatase inhibitors, followed by manual disruption. Resultant supernatants were centrifuged at 16,000 xg for 10 min at 4°C and protein concentration of debris free supernatant was determined using a BCA protein assay kit (Thermo Fisher scientific, Loughborough, UK) following the manufacturer’s protocol. Samples were then diluted 3:1 with 4x laemmli buffer (250 mM Tris-HCl, pH 6.8, 8% SDS, 40% glycerol, 0.004% bromophenol blue, 20% β-mercaptoethanol), heated at 95°C for 5 min and either loaded directly onto an SDS-PAGE gel or placed at -80°C. SDS-PAGE was conducted using a 10% gel and 30 μg total protein per sample. Samples were then transferred onto Hybond ECL nitrocellulose (GE healthcare, Buckinghamshire, UK) and membranes blocked using 5% BSA in TBS-T for 2 hours at RT or overnight at 4°C. After blocking, membranes were incubated with rabbit anti-phospho-ERK1/2, rabbit anti-phospho-Akt or rabbit anti-phospho-p38 (all used at 1:1000), diluted in 5% BSA/TBS-T for 2 hours at RT or overnight at 4°C. Membranes were then incubated with a HRP-conjugated anti-rabbit secondary antibody (1:20,000) for 1 hour at RT. Protein bands were visualised by incubating the membranes for 5 min with Amersham™ ECL prime and subsequent exposure to x-ray film over a range of exposure times. To confirm equal protein loading between samples, bound antibodies were removed by incubating the nitrocellulose membranes in stripping buffer (60 mM Tris-HCl pH 6.8, 2% SDS, 0.8% β-mercaptoethanol) for 30 min at 50°C. Membranes were then blocked with 5% BSA in TBS-T for 2 hours at RT and then incubated with rabbit anti-ERK1/2 or rabbit anti-p38 (both 1:1000) diluted in 5% BSA/TBS-T for 2 hours. Protein band detection was conducted as described above. Densitometric analysis was conducted using Image Studio Lite (LI-COR Biosciences, Cambridge, UK) and the phosphorylated protein band density was divided by total ERK1/2 band density. Data are displayed as a fold change relative to vehicle treated samples. Full, uncropped blots can be found in S5 Fig.

### Real time PCR

RNA extraction from BMDMs or RAW264.7 macrophages was conducted using the RNeasy® Mini Kit (Qiagen, Manchester, UK) following the manufacturer’s instructions. RNA concentration and quality was determined using a NanoDrop™ ND-1000 spectrophotometer. cDNA was subsequently produced using the QuantiTect® Reverse Transcription kit (Qiagen) following the manufacturer’s instructions. Expression of actin or CCL2 was determined using the following primers: (5’>3’): Actin Fwd CCAACAGCAGACTTCCAGGATT, Actin Rev CTGGCAAGAAGGAGTGGTAACTG, CCL2 Fwd CAGCACCTTTGAATGTGAAGTTG, CCL2 Rev TGCTTGAGGTGGTTGTGGAA and SYBR Select PCR master mix (Life Technologies). 500 ng of cDNA was used per reaction with the following thermal profile: 95°C for 5 min, 40 cycles of 95°C for 30 s, 60°C for 20 s, 72°C for 30 s and a final step of 72°C for 5 min using a StepOnePlus™ thermal cycler (Applied Biosystems). Cycle threshold values (Ct) were calculated using StepOne™ software version 2.3 and data are displayed as 2^-ΔCt (referred to as relative to actin) whereby ΔCt = Ct value of gene of interest minus Ct value of actin.

### Data and statistical analysis

All data were analysed using Graphpad Prism version 6. For comparisons between two groups, an unpaired two-tailed student’s t test was applied. For multiple comparisons with only one variable, a one-way ANOVA with Dunnett’s multiple comparisons correction was applied and for multiple comparisons with two variables, a two-way ANOVA with Sidak’s multiple comparisons correction was applied. The exact test applied to each data set is stated in the corresponding figure legends. Data are expressed as mean + or ± SEM, as stated in the figure legend. A P value of < 0.05 was taken to be statistically significant.

## Results

**Fig 1:**
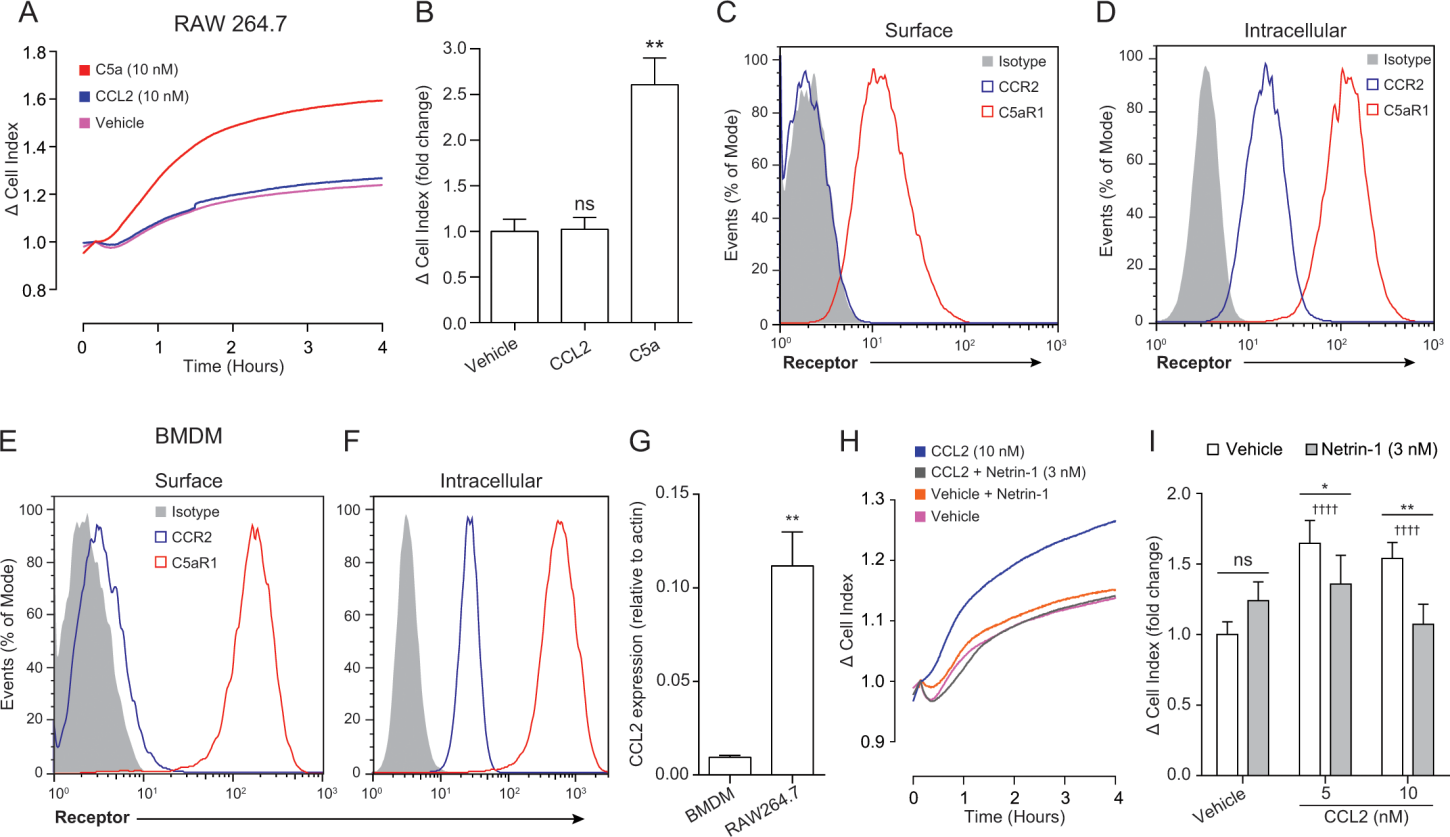
Netrin-1 inhibits primary macrophage chemotaxis towards CCL2. (**A**) RAW264.7 macrophages (1x10^5^) were placed into the top chamber of a CIM-16 plate and allowed to migrate towards vehicle, C5a or CCL2 (both 10 nM) for 4 hours at 37°C, 5% CO_2_. (**B**) Quantification of RAW264.7 chemotaxis by Δ CI analysis. Data are mean + SEM, n = 3 biological replicates with 3–4 technical replicates per condition. RAW264.7 macrophages or BMDMs were stained for (**C** and **E**) surface or (**D** and **F**) intracellular levels of CCR2 and C5aR1 and analysed by flow cytometry. Histograms shown are representative of 3 biological replicates. (**G**) mRNA expression of CCL2 in BMDMs and RAW264.7 macrophages was determined using RT-PCR. Data are mean + SEM, n = 3 biological replicates. (**H**) BMDMs were treated with either vehicle or 3 nM netrin-1 for 45 min at 37°C, 5% CO_2_ prior to being placed into the upper chamber of a CIM-16 plate (1x10^5^ cells/well). They were then allowed to migrate towards for 4 hours at 37°C, 5% CO_2_ towards vehicle or CCL2 at the indicated concentration. (**I**) Quantification of BMDM chemotaxis by Δ CI analysis. Data are mean + SEM, n = 3 biological replicates with 3–4 technical replicates per condition. For (**B**) statistical analysis was conducted by one-way ANOVA with Dunnett’s multiple comparisons correction. ns P > 0.05, ** P < 0.01 compared to vehicle. For (**G**) statistical analysis was conducted by unpaired two-tailed student’s t test. For (**I**) statistical analysis was conducted by two-way ANOVA with Sidak’s multiple comparisons correction. ns P > 0.05, * P < 0.05, ** P < 0.01, for indicated comparisons and †††† P < 0.001, compared to the respective vehicle control.

We began our study by attempting to replicate the finding that netrin-1 is able to inhibit the chemotaxis of the RAW264.7 macrophage cell line towards the CC chemokine, CCL2 [12]. Surprisingly however, we discovered that in our hands RAW264.7 macrophages did not migrate towards CCL2 (Fig 1A and 1B). This was not simply due to the fact that they were unable to migrate, as the anaphylatoxin C5a, which is a potent macrophage chemoattractant, significantly induced RAW264.7 macrophage chemotaxis (Fig 1A and 1B). Analysis of RAW264.7 receptor expression by flow cytometry demonstrated that these cells lacked CCR2, but not C5aR1 (the cognate GPCRs for CCL2 and C5a respectively), on their surface (Fig 1C), whereas both receptors were present intracellularly (Fig 1D). In order to try and replicate previous findings that netrin-1 is capable of inhibiting macrophage chemotaxis to CCL2, we switched to using primary murine bone marrow derived macrophages (BMDMs). Compared to the isotype control, BMDMs stained positively for surface CCR2 expression (Fig 1E) although clearly the majority of the BMDM CCR2 expression was intracellular (Fig 1F). Real-time PCR analysis showed that RAW264.7 macrophages expressed twelve times more CCL2 mRNA than BMDMs (Fig 1G). This potentially explains the lack of surface CCR2 on RAW264.7 macrophages in comparison to BMDMs, as the higher autocrine CCL2 production by RAW264.7 macrophages will likely cause greater desensitisation and internalisation of CCR2. Mirroring the results obtained with the RAW264.7 macrophages, BMDMs had high surface and intracellular levels of C5aR1 (Fig 1E and 1F). Real-time chemotaxis analysis demonstrated that BMDMs migrated towards CCL2 at 5 and 10 nM (Fig 1H and 1I) and that pre-treatment with 3 nM netrin-1 significantly reduced BMDM chemotaxis towards CCL2 at 5 and 10 nM (Fig 1I). Netrin-1 was used at a concentration of 3 nM based on our preliminary experiments and the fact that the maximal inhibitory effect of netrin-1 on macrophage chemotaxis has been observed at this concentration [5, 12].

As the surface expression of CCR2 seemed low on BMDMs, we were interested to compare this with other primary macrophage populations. We selected bio-gel elicited macrophages, as we have previously shown that they migrate towards CCL2 in our real-time chemotaxis system [2]. Flow cytometric analysis demonstrated that bio-gel elicited macrophages had a small but statistically significant amount of CCR2 on their cell surface, comparable to that of BMDMs (S1A–S1E Fig), and real-time chemotaxis analysis confirmed that these primary macrophages were capable of migrating towards 5 nM CCL2 (S1F Fig). In agreement with all macrophage populations tested so far, bio-gel elicited macrophages had high surface C5aR1 levels (S1A–S1E Fig).

**Fig 2:**
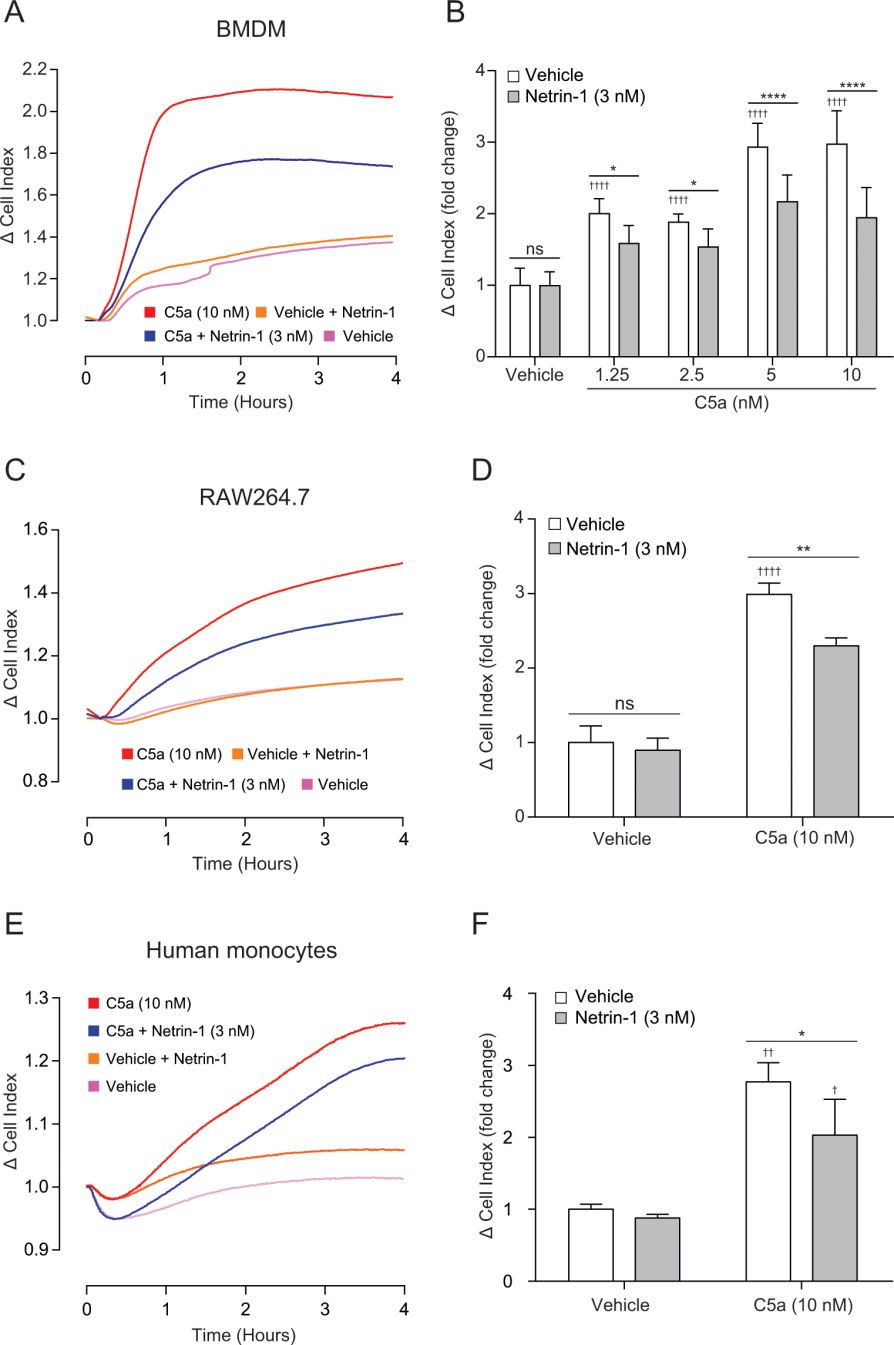
Netrin-1 inhibits primary murine macrophage and human monocyte chemotaxis towards the complement peptide C5a. (**A**) BMDMs were incubated with either vehicle or 3 nM netrin-1 for 45 min at 37°C, 5% CO_2_ prior to being placed into the upper chamber of a CIM-16 plate (1x10^5^ cells/well). Migration was then measured for 4 hours at 37°C, 5% CO_2_ towards vehicle or C5a at the indicated concentration. (**B**) Quantification of BMDM chemotaxis by Δ CI analysis. Data are mean + SEM, n = 5 biological replicates with 3–4 technical replicates per condition. (**C**) RAW264.7 macrophages were incubated with either vehicle or 3 nM netrin-1 for 45 min at 37°C, 5% CO_2_ prior to being placed into the upper chamber of a CIM-16 plate (1x10^5^ cells/well). Migration was then measured for 4 hours at 37°C, 5% CO_2_ towards vehicle or 10 nM C5a. (**D**) Quantification of RAW264.7 chemotaxis by Δ CI analysis. Data are mean + SEM, n = 4 biological replicates with 3–4 technical replicates per condition. (**E**) Human CD14^+^ monocytes were treated with either vehicle or 3 nM netrin-1 for 45 min at 37°C, 5% CO_2_ prior to being placed into the upper chamber of a CIM-16 plate (1x10^5^ cells/well). They were then allowed to migrate towards for 4 hours at 37°C, 5% CO_2_ towards vehicle or 10 nM C5a. (**F**) Quantification of human monocyte chemotaxis by Δ CI analysis. Data are mean + SEM, n = 3 biological replicates with 3–4 technical replicates per condition. Statistical analysis was conducted by two-way ANOVA with Sidak’s multiple comparisons correction. ns P > 0.05, * P < 0.05, ** P < 0.01, **** P < 0.0001 for indicated comparisons. † P < 0.05, †† P < 0.01, †††† P < 0.0001, compared to the respective vehicle.

Given that our main objective with this study was to determine whether netrin-1 is capable of inhibiting macrophage chemotaxis to non-chemokine chemoattractants, and that all macrophage populations we tested had high surface levels of C5aR1, we next examined the effect of netrin-1 on C5a induced macrophage chemotaxis. Treatment of BMDMs with 3 nM netrin-1 for 45 minutes inhibited their migration towards 1.25, 2.5, 5 and 10 nM C5a (Fig 2A and 2B), whereas netrin-1 had no effect on basal BMDM migration in this assay (Fig 2A and 2B). The same inhibitory effect was also observed when using RAW264.7 macrophages, as netrin-1 treatment significantly reduced their migration towards C5a (Fig 2C and 2D). To further extend our observation, we next tested the ability of netrin-1 to inhibit human monocyte chemotaxis as these cells are critical for the initiation and continuation of multiple inflammatory diseases [4]. Real-time chemotaxis analysis demonstrated that netrin-1 significantly inhibited human monocyte chemotaxis towards C5a, with the size of reduction comparable to that seen in the murine macrophages (~ 30% reduction; Fig 2E and 2F). Taken together, these experiments demonstrate that netrin-1 is capable of inhibiting monocyte-derived cell migration towards C5a.

**Fig 3:**
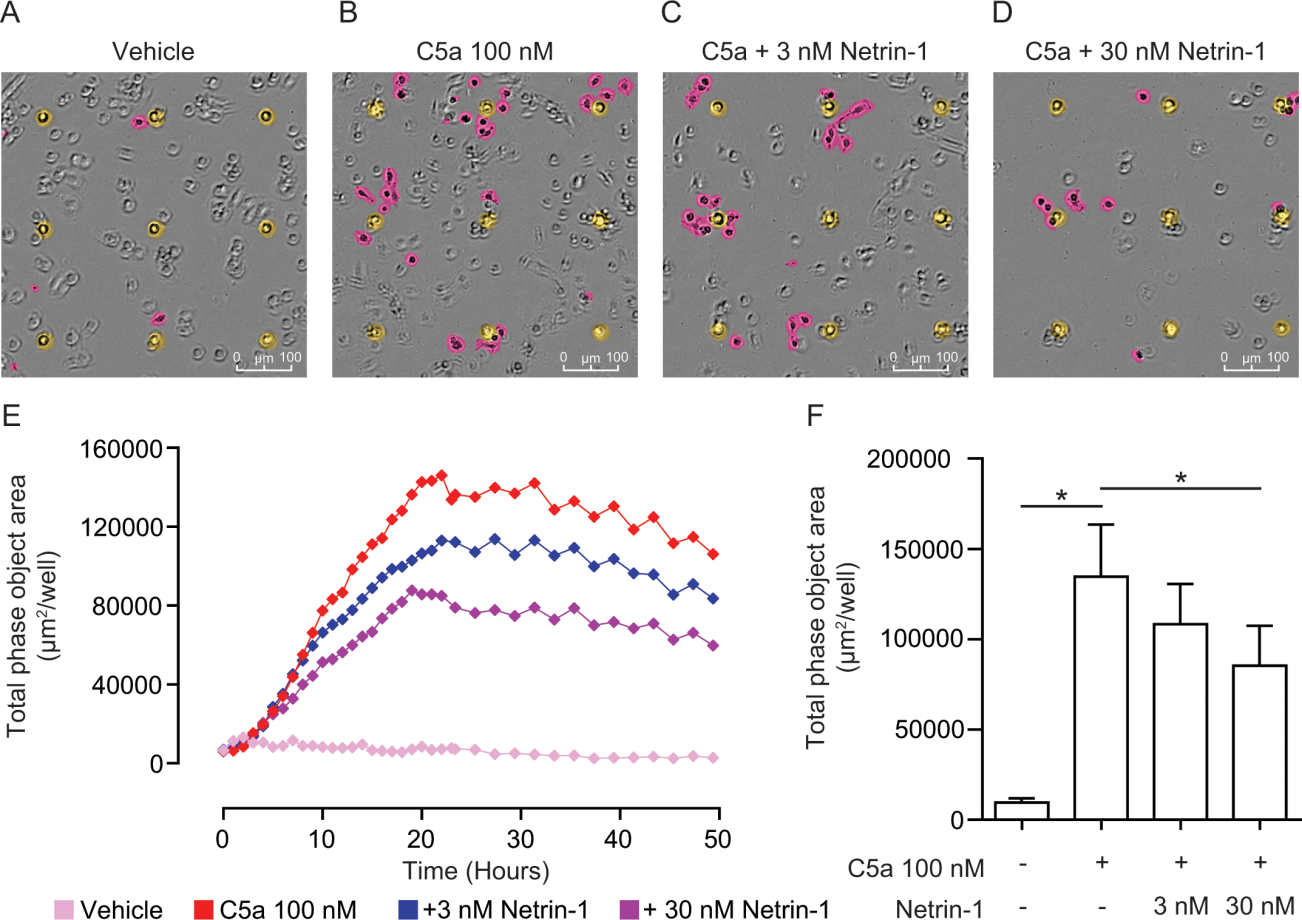
Confirmation of the inhibitory effects of netrin-1 on macrophage chemotaxis using a real-time microscopy based migration assay. BMDMs were incubated with either vehicle, 3 nM or 30 nM netrin-1 for 45 min at 37°C, 5% CO_2_ prior to being placed into the upper chamber of an IncuCyte® ClearView chemotaxis plate (5000 cells/well), which had been coated with Matrigel (50 μg/ml for 30 min at 37°C and 30 min at RT). Cells were then allowed to migrate towards vehicle or 100 nM C5a and chemotaxis was measured by imaging the underside of the well every 2 hours and calculating total underside cell area. Representative images of cells migrating towards (**A**) vehicle or (**B**) 100 nM C5a and cells migrating towards 100 nM C5a that had been treated with (**C**) 3 nM or (**D**) 30 nM netrin-1. The pores connecting the upper and lower chambers are masked in yellow and cells that have migrated to the underside of the well are masked in pink. (**E**) A representative real-time trace of total cell area on the underside of the well against time. (**F**) Quantification of BMDM chemotaxis by maximum cell area reached on the underside of the well. Data are mean + SEM, n = 4 biological replicates with 4 technical replicates per condition. Statistical analysis was conducted by one-way ANOVA with Dunnett’s multiple comparisons correction.* P < 0.05 for indicated comparisons.

In order to corroborate our real-time chemotaxis results obtained using the ACEA xCELLigence system, we decided to employ a microscopy based assay that allows measurement of cell migration in real-time. The IncuCyte® ZOOM system uses custom Boyden-chamber style 96 well plates, where each well contains 96 8 μm diameter fixed location pores in the filters that separate the upper and lower halves. The microscope takes images of the topside and the underside of each filter and the analysis software then identifies the location of each pore and cells in focus on the top and the bottom of the filter in each well. As can be seen in the representative underside images in Fig 3A–3D, identified pores have been masked yellow by the software, whereas cells in focus on the underside (i.e. cells that have migrated from the top to the bottom) are masked in pink. Total surface area covered by cells that have migrated to the underside is calculated by the software and provides a measure of chemotaxis. Out of focus cells on the topside can still be clearly seen.

In preliminary experiments, we found that higher C5a concentrations than those used in our impedance based real-time chemotaxis system were needed to cause robust BMDM chemotaxis, and therefore 100 nM C5a was chosen as an optimal concentration (S2A Fig). In comparison to cells migrating towards vehicle alone, where almost no migration was detected (Fig 3A, 3E and 3F), 100 nM C5a induced a significant increase in the total phase object area on the underside of the well (Fig 3B (pink, in focus objects), 3E and 3F). Treatment of BMDMs with 3 nM netrin-1 caused a slight reduction in cell migration (Fig 3C, 3E and 3F), whereas 30 nM netrin-1 significantly blocked C5a induced chemotaxis (Fig 3D, 3E and 3F). The magnitude of inhibition was very similar to our previous results (~30% reduction). We ruled out unequal cell loading as a potential explanation for the difference in migration observed with netrin-1 treatment, as we found that there was no difference in the total topside area covered by cells at time point zero for each condition (S2B Fig). Collectively this real-time chemotaxis data, obtained using a microscopy based method, corroborates our finding that netrin-1 significantly inhibits macrophage chemotaxis induced by C5a, but does not reduce it to baseline.

**Fig 4:**
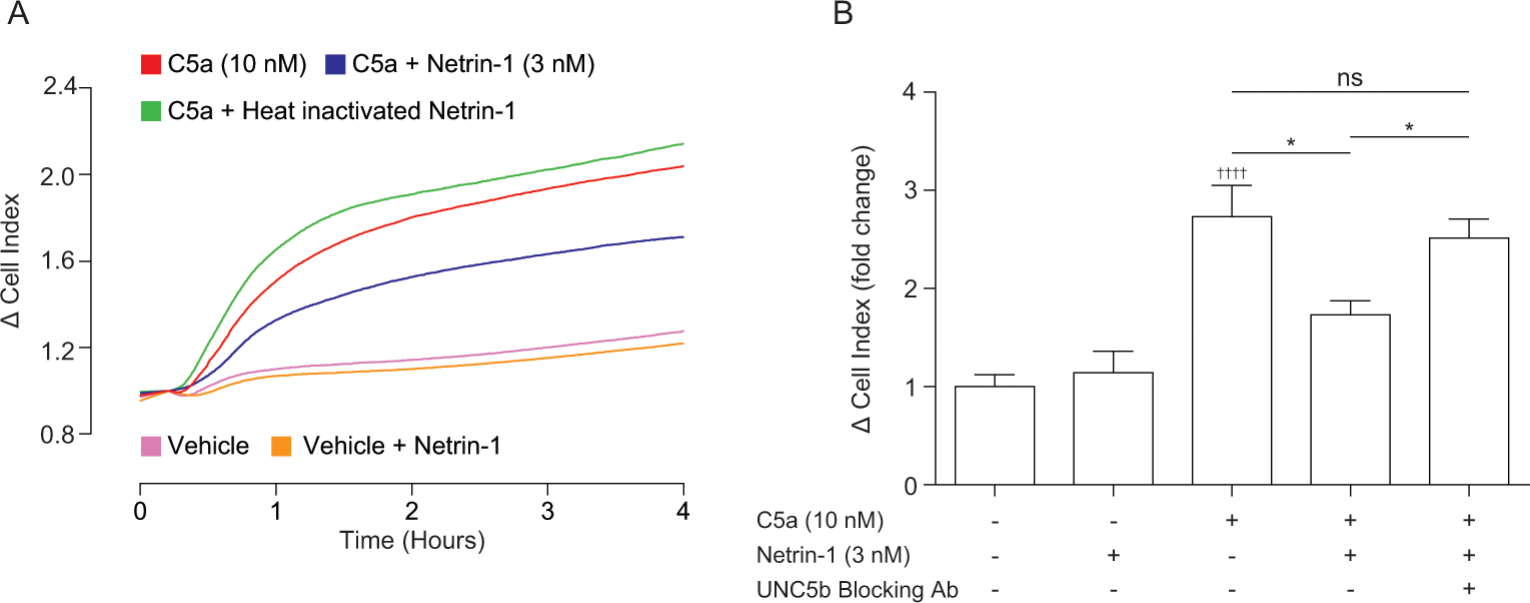
Activation of UNC5b by fully folded netrin-1 is required for inhibition of C5a induced primary macrophage chemotaxis. (**A**) BMDMs were incubated with either vehicle, 3 nM netrin-1, or 3 nM heat inactivated netrin-1 (heated to 75°C for 25 min) for 45 min at 37°C, 5% CO_2_ prior to being placed into the upper chamber of a CIM-16 plate (1x10^5^ cells/well). They were then allowed to migrate towards for 4 hours at 37°C, 5% CO_2_ towards vehicle or 10 nM C5a. (**B**) BMDMs were incubated with either vehicle, 3 nM netrin-1 alone or 3 nM netrin-1 in the presence of an UNC5b blocking antibody (10 μg/ml) for for 45 min at 37°C, 5% CO_2_ prior to being placed into the upper chamber of a CIM-16 plate (1x10^5^ cells/well). Cells were then allowed to migrate for 4 hours at 37°C, 5% CO_2_ towards vehicle or 10 nM C5a. Chemotaxis was quantified by Δ CI analysis. Data are mean + SEM, n = 4 biological replicates with 3–4 technical replicates per condition. Statistical analysis was conducted by one-way ANOVA with Dunnett’s multiple comparisons correction. ns P > 0.05, * P < 0.05, for indicated comparisons. †††† P < 0.0001, compared to the respective vehicle control.

To begin to understand how netrin-1 inhibits macrophage chemotaxis, we first tested whether netrin-1 needed to be in its native conformation to reduce cellular migration. To do this, we denatured netrin-1 by heating to 75°C for 25 minutes before BMDM treatment. In contrast to non-denatured netrin-1, which inhibited BMDM chemotaxis (Fig 4A–blue line), heat inactivated netrin-1 had no effect on C5a induced BMDM chemotaxis (Fig 4A–red vs green lines). Following this, we wanted to examine the dependence of the inhibitory effect of netrin-1 on netrin receptor signalling. We focussed on UNC5b as the UNC family members are the only netrin receptors reported to be expressed on leukocytes [21] and UNC5b signalling has previously been demonstrated to be responsible for the inhibitory effects of netrin-1 on chemokine induced macrophage chemotaxis [5, 12]. We tested reliance on UNC5b signalling using an UNC5b blocking antibody. As previous, treatment of BMDMs with netrin-1 significantly reduced their migration towards C5a (Fig 4B), however co-incubation of BMDMs with netrin-1 and an UNC5b blocking antibody significantly reversed the inhibitory effect of netrin-1 (Fig 4B). Finally, we examined the effect of netrin-1 treatment on cell viability. We found that treatment with 3 or 30 nM netrin-1 for 45 minutes, 4 hours or even 24 hours had no effect on cell viability (S3A Fig), ruling out induction of cell death as an explanation for inhibition of cell migration by netrin-1. Taken together, these data demonstrate that netrin-1 activates its cognate receptor UNC5b to inhibit macrophage chemotaxis towards C5a.

**Fig 5:**
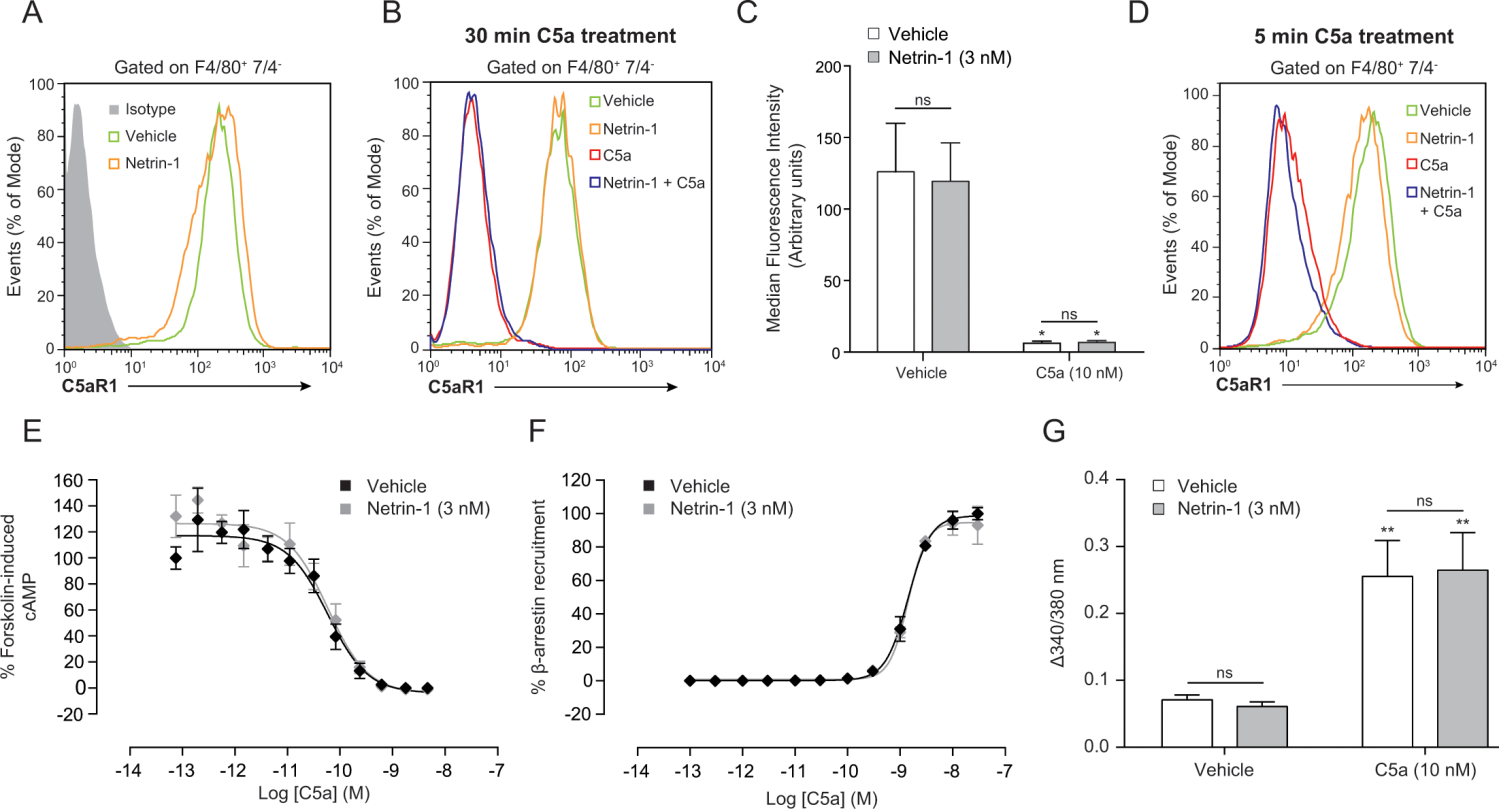
Netrin-1 does not inhibit C5aR1 proximal signalling events. BMDMs were incubated with either vehicle of 3 nM netrin-1 for 45 min at 37°C, 5% CO_2_ prior to being stimulated with either vehicle or 10 nM C5a for the time indicated. Cells were then subjected to antibody staining for surface levels of F4/80, 7/4 and C5aR1. Representative flow cytometry histograms of BMDM (defined as F4/80^+^, 7/4^-^) C5aR1 surface levels following stimulation for 30 min with either vehicle or 10 nM C5a, with and without 3 nM netrin-1 treatment are shown in (**A**) and (**B**) respectively. (**C**) Quantification of C5aR1 surface levels by median fluorescence intensity. Data are mean + SEM, n = 3 biological replicates. (**D**) Flow cytometry histogram showing BMDM surface C5aR1 levels following 5 min C5a (10 nM) stimulation, with and without netrin-1 (3 nM) treatment. (**E**) CHO-K1 cells expressing human C5aR1 were incubated with either vehicle or 3 nM netrin-1 for 45 min at 37°C, 5% CO_2_. Cells were then stimulated with the indicated concentration of C5a for 30 min at 37°C, 5% CO_2_ and intracellular cAMP levels measured. (**F**) CHO-K1 cells expressing murine C5aR1 were incubated with either vehicle or 3 nM netrin-1 for 45 min at 37°C, 5% CO_2_. Cells were then stimulated with the indicated concentration of C5a for 90 min at 37°C, 5% CO_2_ and β-arrestin recruitment to C5aR1 measured. Data are mean ± SEM, n = 3 technical replicates. (**G**) BMDMs were seeded into 96 well black walled plates (50,000 cells/well) and incubated with 4 μM Fura 2-AM with or without 3 nM netrin-1 for 45 min at RT. Cells were then washed, the medium replaced and then stimulated with either vehicle or 10 nM C5a. Change in Fura-2 fluorescence at 340 nm/510 nm and 380 nm/510 nm was calculated. Data are mean + SEM, n = 3 biological replicates with 2 technical replicates per condition. Statistical analysis was conducted by two-way ANOVA with Sidak’s multiple comparisons correction. ns P > 0.05, for indicated comparisons and * P < 0.05, ** P < 0.01, compared to the respective vehicle control.

In our search for the inhibitory mechanism of netrin-1, we initially focused on C5aR1 proximal signalling events. As our macrophage populations had high surface expression of C5aR1, this put us in the unique position of being able to accurately measure changes in macrophage C5aR1 surface levels and dynamics following netrin-1 exposure. As determined by flow cytometry, treatment of BMDMs with netrin-1 had no effect on basal C5aR1 surface levels (Fig 5A and 5C). Stimulation of BMDMs with C5a for 30 minutes induced the robust internalisation of C5aR1; however pre-treatment with netrin-1 had no effect on receptor internalisation following C5aR1 ligation (Fig 5B and 5C). Even after only 5 minutes of C5a stimulation the vast majority of C5aR1 was absent from the cell surface and netrin-1 had no effect on this rapid internalisation of C5aR1 (Fig 5D). Next we wanted to assess whether netrin-1 had any effect on classical signalling pathways immediately proximal to G_i/o_-coupled GPCR activation. In CHO cells expressing C5aR1, C5a induced a concentration dependent decrease in forskolin-induced intracellular cAMP levels and a concentration dependent increase in β-arrestin recruitment to C5aR1, as would be expected. However, netrin-1 had no effect on either of these signalling pathways (Fig 5E and 5F, respectively). Additionally, netrin-1 had no effect on basal Ca^2+^ handling in BMDMs, and C5a induced increases in intracellular Ca^2+^ were not affected by netrin-1 treatment, demonstrating that Ca^2+^ signalling remained intact (Fig 5G). Taken together, these results show that netrin-1 does not affect receptor proximal signalling events that are initiated following C5aR1 engagement.

**Fig 6:**
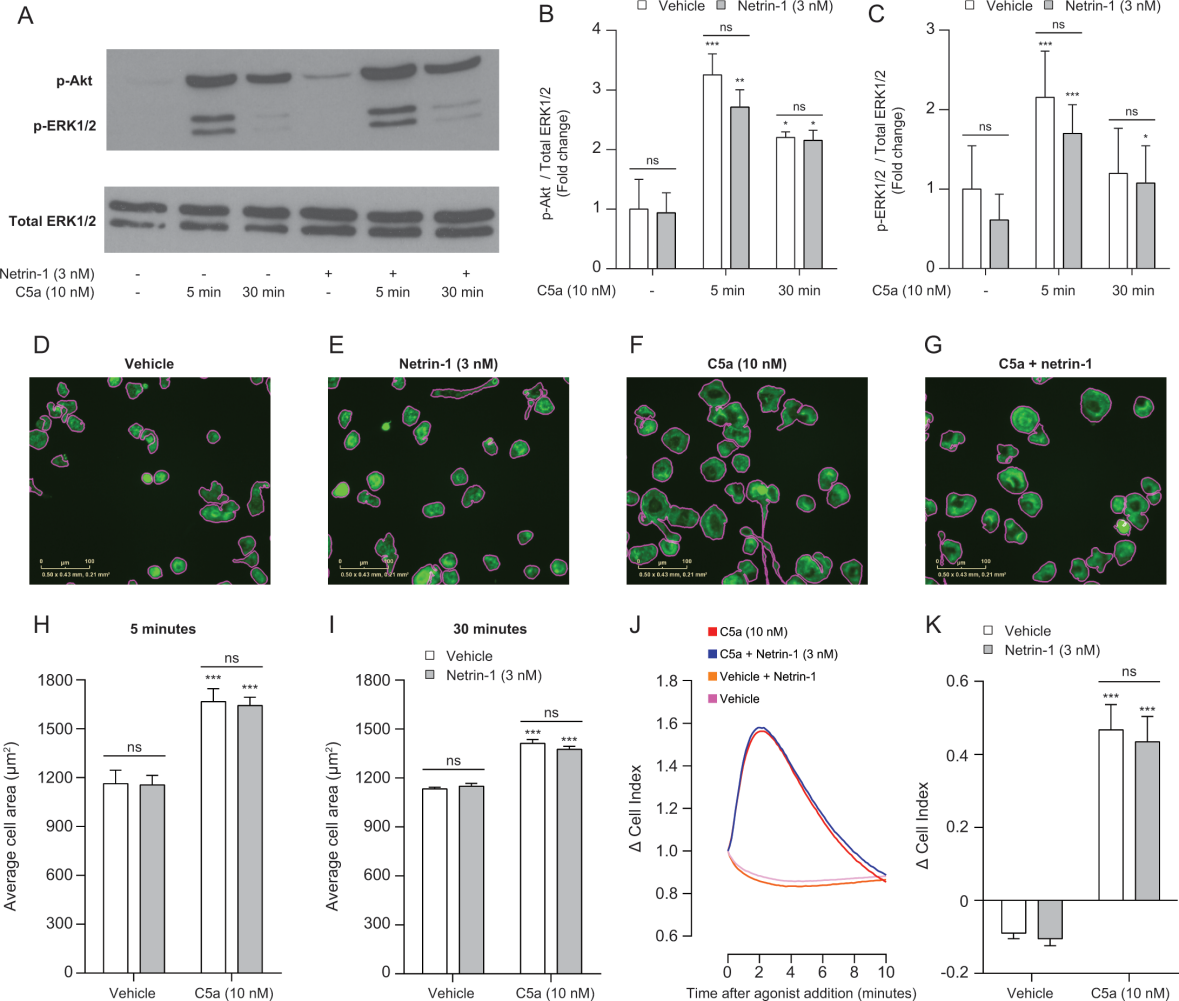
Netrin-1 does not affect distal signalling events following C5aR1 ligation. (**A**) BMDMs were seeded into 6 well dishes (2x10^6^ cells/dish) and then treated with either vehicle or 3 nM netrin-1 for 45 min at 37°C, 5% CO_2_. Cells were then stimulated with either vehicle or 10 nM C5a for 5 or 30 min at 37°C, 5% CO_2_. Cell lysates were prepared and western blotting conducted for either phosphorylated Akt (p-Akt) or ERK1/2 (p-ERK1/2), followed by stripping and re-staining for total ERK1/2 as a loading control. Quantification by densitometry of C5a-induced (**B**) Akt or (**C**) ERK1/2 phosphorylation. Data are mean + SEM, n = 3 biological replicates. BMDMs were seeded into 96 well black walled plates (5000 cells/well) and then treated with either vehicle or 3 nM netrin-1 for 45 min at 37°C, 5% CO_2_. Cells were then stimulated with either vehicle or 10 nM C5a for 5 or 30 min before being fixed and stained with Alexa Fluor® 488 phalloidin to visualise F-actin. Images were then taken using an IncuCyte® ZOOM microscope and the average cell area calculated. Representative images showing phalloidin staining in green and the cell outlines calculated by the IncuCyte® ZOOM software in pink for cells stimulated with (**D**) vehicle alone, (**E**) 3 nM netrin-1 alone, (**F**) 5 min 10 nM C5a alone and (**G**) 3 nM netrin-1 and 5 min 10 nM C5a. Quantification of average cell area after (**H**) 5 min or (**I**) 30 min stimulation. Data are mean + SEM, n = 3–5 biological replicates with 4 technical replicates per condition. (**J**) BMDMs (2 x 10^4^) were placed into a 96 well E-plate allowed to adhere for 2–3 hours at 37°C, 5% CO_2_. Afterwards, cells were treated with either vehicle or 3 nM netrin-1 for 45 min prior to being stimulated with vehicle or 10 nM C5a. (**K**) Quantification of the change in cell index following stimulation. Data are mean + SEM, n = 4 biological replicates with 3–4 technical replicates per condition. Statistical analysis was conducted by two-way ANOVA with Sidak’s multiple comparisons correction. ns P > 0.05, for indicated comparisons. * P < 0.05, ** P < 0.01, *** P <0.001 compared to the respective vehicle control.

To continue the search for a potential mechanism, next we assessed signalling events that lie further downstream of C5aR1 activation. We measured ERK1/2 and Akt phosphorylation following netrin-1 and C5a treatment in BMDMs, as the former has been implicated in regulating chemotaxis and the latter is a surrogate marker for PI3K activity, a critical pathway for directed cell migration [22]. C5a stimulation caused a rapid and significant increase in the phosphorylation of both ERK1/2 and Akt, which was detectable by 5 minutes (Fig 6A, 6B and 6C). By 30 minutes, ERK1/2 phosphorylation had returned to baseline levels, whereas Akt phosphorylation remained (Fig 6A, 6B and 6C). Nevertheless, netrin-1 treatment had no effect on ERK1/2 or Akt phosphorylation basally or after C5a stimulation at any time point examined (Fig 6A, 6B and 6C). We next examined the effect of netrin-1 on p38 phosphorylation, as p38 MAPK has been previously demonstrated to be important for controlling C5a-induced neutrophil [23] and macrophage [24] chemotaxis. p38 phosphorylation was induced rapidly and transiently in BMDMs following C5a stimulation, as signal was detectable after 5 minutes but had returned to baseline after 30 minutes of C5a treatment. Mirroring the results obtained with both Akt and ERK1/2, netrin-1 had no effect on C5a-induced p38 phosphorylation basally or after C5a treatment (S4A Fig).

Finally as netrin-1 had previously been demonstrated to inhibit CCL2 induced macrophage cytoskeletal rearrangement [12], we measured the effect of netrin-1 on actin dynamics in BMDMs following C5a stimulation. To do this, we used phallodin staining of F-actin combined with automated analysis of BMDM cell area to quickly and accurately calculate average cell area. In an operator independent manner, the average cell area was calculated from approximately 800 cells per condition, greatly increasing the confidence of the results obtained. In the representative images shown in Fig 6D–6G, the pink outlines around the cells are the software calculated cell bounds used for area analysis. In comparison to vehicle treated cells, stimulation of BMDMs for 5 minutes with 10 nM C5a caused rapid cell spreading (Fig 6D, 6F and 6H) and by 30 minutes, this increase in average cell area was still present (Fig 6I). Interestingly, treatment with netrin-1 was unable to inhibit C5a induced BMDM spreading at any time point measured (Fig 6G, 6H and 6I). In an attempt to corroborate this finding, we employed an electrical impedance assay that allows measurement of cytoskeletal dynamics in real-time following GPCR activation [20]. Stimulation of BMDMs with C5a caused a rapid and robust increase in Cell Index, which correlates with increased cell spreading. However, netrin-1 pre-treatment had no effect on the kinetics of C5a induced changes in Cell Index (Fig 6J and 6K), confirming that it is unable to inhibit changes in BMDM morphology following C5a stimulation.

## Discussion

In this study we set out to explore the immunomodulatory role of netrin-1 on macrophage chemotaxis. Our aims were twofold: to reproduce the published finding that netrin-1 can reduce macrophage chemotaxis towards CCL2 [12], and to extend the panel of chemoattractants towards which netrin-1 can be shown to modulate macrophage chemotaxis. Using two different real-time chemotaxis methodologies, we report for the first time that netrin-1 reduces human monocyte and murine macrophage migration towards the complement component C5a. We observe a partial (~30%) yet significant reduction in chemotaxis towards C5a in an UNC5b dependent manner and demonstrate that changes in C5aR1 internalisation, PI3K/MAPK signalling, Ca^2+^ release, and cell spreading are not responsible for the observed inhibition.

The choice of macrophage population was an important consideration throughout our investigation. van Gils *et al* previously reported that RAW264.7 macrophages migrate towards CCL2 and that netrin-1 abolishes this response [12]. However, our own RAW264.7 macrophages did not migrate towards CCL2, likely due to the fact that they did not have surface expression of CCR2. This contrasted with both BMDMs and Bio-gel elicited macrophages which had a small surface CCR2 population that was sufficient to facilitate their migration towards CCL2. As it has been previously shown that RAW264.7 macrophages constitutively produce CCL2 [25] and we found that that RAW264.7 macrophages express 12 times more CCL2 than BMDMs, we believe that the higher level of CCL2 produced by RAW264.7 macrophages acts in an autocrine manner to cause greater desensitisation and internalisation of CCR2, a known consequence of prolonged chemokine receptor activation [26]. This therefore explains why CCR2 expression could only be detected intracellularly in RAW264.7 macrophages and why BMDMs, but not RAW264.7 macrophages, migrate towards CCL2. Why our RAW264.7 cell line behaves differently to others is not currently clear, although potentially this discrepancy could be the result of genetic drift. Both time in culture and culture conditions used can lead to genetic changes that impact on the phenotype of the cell line [27], and others have shown that RAW264.7 subclones can display markedly different behaviour to that of the parental line [28, 29].

Nonetheless, BMDMs were previously unstudied in the setting of macrophage chemotaxis with netrin-1 and we observed that their migration towards CCL2 was inhibited by netrin-1 treatment, corroborating the inhibitory effect of netrin-1 on chemokine-induced chemotaxis. However, all macrophage populations we used in this study had high surface expression of C5aR.1 This prompted our investigation into C5a induced macrophage chemotaxis and led to the novel discovery that this could be significantly reduced by netrin-1 in RAW264.7 macrophages, BMDMs, and human monocytes.

We believe that one advantage of our study over previously published work is the fact that we use real-time methodologies to quantify cell migration. Currently, Boyden chamber style migration assays are predominantly used to study chemotaxis of multiple cell types. However, these only afford the measurement of cell migration at a single time point, and as the kinetics of cell migration vary markedly depending on the cell type studied and the chemoattractant used, this limits the utility of this type of assay. For example, a study performed by Maiuri *et al* compared the migration of 54 different cell types under standardised conditions and found that there was a large variation in migration speed between the cells tested [30]. Furthermore, we have previously shown that the kinetics of human monocyte migration varies between donors in the xCELLigence system and that after peak migration is seen the signal drops dramatically towards the end of the assay, likely due to the cells detaching from the underside of the filter [2]. This therefore highlights that there can be large inter and intra cell variability of migration kinetics and means that any single time point analysis of chemotaxis may miss true chemotactic responses, potentially resulting in false positive or negative conclusions about differences in chemotaxis between cell types or treatments. However, it should be noted that real-time chemotaxis assays are not without their disadvantages. For example, the xCELLigence system cannot measure the chemotaxis of non-adherent cell types and the IncuCyte® ZOOM seems to require higher chemoattractant concentrations when compared to other migration assays. Nonetheless, by using two different real-time chemotaxis assays, namely the ACEA xCELLigence (electrical impedance) and IncuCyte® ZOOM (microscopy) systems, we are confident in our conclusion that netrin-1 inhibits C5a induced macrophage chemotaxis. Both systems concur on the degree of inhibition by netrin-1, as well as having very similar relative chemotaxis kinetics. We hope that in the future, when examining chemotaxis, that scientists will endeavour to utilise real-time chemotaxis methods, alongside Boyden chamber assays, to maximise the reliability of any inferences drawn from their data.

In all studies published to date, netrin-1 has always fully inhibited cellular migration towards a range of chemoattractants and indeed, we were able to confirm previous results by demonstrating that netrin-1 fully inhibited CCL2 induced BMDM chemotaxis. Unexpectedly however, and in stark contrast to this, we found that netrin-1 never fully blocked C5a induced chemotaxis; instead it only ever inhibited migration by around 30%. We speculate that that this discrepancy may be caused by macrophages having much higher surface levels of C5aR1 than chemokine receptors, which results in stronger downstream chemotactic signalling following C5aR1 ligation that is more difficult for netrin-1 induced inhibitory signalling to overcome. In all macrophage populations examined, C5aR1 was expressed at much higher levels on the cell surface than CCR2 and C5a was capable of inducing a much larger chemotactic response than CCL2 (~3 fold increase vs ~1.5 fold increase, respectively), demonstrating that chemotactic drive seemed to be correlated with surface receptor expression. Further support for this hypothesis comes from our cell spreading experiments. In contrast to van Gils *et al* who show that netrin-1 is capable of fully inhibiting CCL2 induced macrophage spreading [12], we found that netrin-1 had no effect on macrophage shape change following C5aR1 ligation using two different assays. Macrophage spreading occurs rapidly when cells are exposed to a chemoattractant gradient and prior to the establishment of front/rear cell polarisation essential for directional migration [3]. As netrin-1 is able to fully inhibit CCL2 induced macrophage chemotaxis, it is reasonable to expect full inhibition of actin polymerisation needed for chemotaxis. However, as macrophage migration towards C5a following netrin-1 treatment was only blunted, we hypothesise that the macrophage remains able to respond to the chemoattractant signal, but cannot fully perform the actin rearrangements needed for efficient directed migration. This therefore results in normal early cell spreading, but impaired directional migration.

In the search for the mechanism of action of netrin-1, we performed a proximal to distal analysis of the effect of netrin-1 on C5aR1 dynamics and signalling; a first in the field. A direct competitive effect of netrin-1 at C5aR1 was ruled out, as C5a induced cAMP signalling and β-arrestin recruitment remained unaffected by netrin-1 in C5aR1 expressing CHO cells. In BMDMs we also ruled out the hypothesis that netrin-1 effected receptor dynamics, as surface C5aR1 levels or its internalisation remained unchanged following netrin-1 treatment. Furthermore, netrin-1 had no effect on proximal receptor signalling following C5aR1 ligation, including the PI3K pathway that is central to cell migration [22]. In light of these findings, we believe that netrin-1 acts at a downstream target needed, but not critical, for chemotaxis. Indeed, the fact that netrin-1 inhibits cell migration to multiple chemoattractants provides further evidence that a peripheral regulator of migration is likely involved [5, 12]. It has been reported that netrin-1 inhibits CCL2 induced activation of Rac-1 [12], providing the hypothesis that small GTPases involved in actin rearrangement may be a target for netrin-1. Rac-1, Cdc42 and Rho belong to the Rho family of small GTPases and regulate actin dynamics; Rac-1 is needed for the formation of lamellipodia, Cdc42 for filopodia formation, and Rho for cell contraction [31]. All three are required for directed migration, as the injection of inhibitors or dominant negative isoforms of these proteins into macrophages results in a loss of efficient chemotaxis and gradient sensing [3, 32, 33]. Interestingly, recent work found that in fibroblasts, Rac-1 is needed for cell migration but not cell spreading [34]. If Rac-1 is inhibited by netrin-1 in macrophages, this could explain why C5a induced macrophage spreading remains intact, but chemotaxis is impaired. However, as netrin-1 can fully inhibit CCL2 induced macrophage spreading, this suggests that Rac-1 may not be the only GTPase target for netrin-1. Furthermore, it is likely that the degree of inhibition of cell migration depends on the balance between positive chemotactic signalling and inhibitory netrin-1 signalling. What is clear is that further work is needed to fully understand the effect of netrin-1 signalling on the function of actin reorganising GTPases.

Regardless of the precise mechanism, the finding that netrin-1 inhibits C5a induced monocyte and macrophage chemotaxis uncovers a novel area of clinical interest. Currently, the therapeutic potential of netrin-1 has largely centred on cardiovascular disease with debate surrounding whether netrin-1 contributes to, or reduces atherosclerotic plaque formation [35]. This conflicting evidence may be explained by the spatial and temporal regulation of netrin-1 secretion. Endothelial-derived netrin-1 acts to inhibit monocyte influx into the arterial intima [36], whereas macrophage foam cell derived netrin-1 inhibits macrophage migration out of fatty lesions [12]. In both situations, cell migration is repressed, but the context determines whether this results in a pro or anti-atherogenic outcome. Furthermore, it has been shown that endothelial cells secrete less netrin-1 in response to pro-inflammatory stimuli [36, 37]. Therefore, as lesions progress, the balance shifts from endothelial-derived netrin-1 to foam cell-derived netrin-1 resulting in a perpetual increase in monocyte recruitment and decrease in foam cell egress.

This context dependent regulation of netrin-1 has direct impact for our present study and the discussion of whether netrin-1 may have therapeutic value in modulating the host complement response. Complement driven pathology is characterised by excessive or inappropriate activation of complement and can manifest as a multitude of inflammatory diseases including sepsis [38]. Peptidomimetic antagonists and allosteric modulators of C5aR1 have shown promising results in experimental models of inflammation [39–42] but have had limited success in clinical trials [43]. The monoclonal antibody Eculizumab inhibits cleavage of C5 to C5a and C5b and is an effective treatment for patients with paroxysmal nocturnal haemoglobinuria (PNH) or atypical haemolytic uremic syndrome (aHUS) [44, 45]. However, Eculizumab treatment is currently limited to PNH and aHUS and treatment costs £340,200 per patient per year [46]. With C5a directly implicated in an increasing number of diseases [47], developing alternative methods to target the complement cascade is crucial and our observations make netrin-1 a viable therapeutic candidate. The incomplete inhibition of chemotaxis towards C5a may also prove to be of higher clinical utility than complete blockade in order to dampen inflammation but preserve complement peptide action in the innate immune response and minimise the risk of bacterial infection [48]. However, this is the first report demonstrating that netrin-1 modulates complement driven chemotaxis and further studies are required to elucidate the extent of netrin-1’s action throughout complement pathways. Moreover, as highlighted above, a more thorough knowledge of the spatiotemporal regulation of netrin-1 is required in order to target this system for maximal therapeutic benefit *in vivo*.

## Supporting Information

S1 Fig Bio-gel elicited macrophages have similar surface levels of CCR2 and C5aR1 to that of bone marrow derived macrophages. Bio-gel elicited macrophages were stained for surface expression of CCR2 and C5aR1. (**A**-**C**) Representative histograms of surface receptor levels, for 3 independent biological samples, compared to isotype control. Quantification of GPCR surface levels by (**D**) median fluorescence intensity or (**E**) geometric mean of fluorescence intensity. Data are mean + SEM, n = 3 biological replicates. (**F**) Bio-gel elicited macrophages were placed into the upper chamber of a CIM-16 plate (2x10^5^ cells/well) and allowed to migrate for 4 hours at 37°C, 5% CO_2_ towards vehicle or 5 nM CCL2. Statistical analysis was conducted by one-way ANOVA with Dunnett’s multiple comparisons correction.* P < 0.05, ** P < 0.01, compared to isotype control.
(EPS)

S2 Fig Optimisation of C5a concentration and demonstration of equal cell loading in the IncuCyte® ZOOM chemotaxis assay. (**A**) BMDMs were placed into the upper chamber of an IncuCyte® ClearView chemotaxis plate (5000 cells/well), which had been coated with Matrigel (50 μg/ml for 30 min at 37°C and 30 min at RT). Cells were then allowed to migrate towards vehicle, 100, 50 or 25 nM C5a and chemotaxis was measured by imaging the underside of the well every 2 hours and calculating total underside cell area. Data are mean of 3–4 technical replicates per condition. (**B**) BMDMs were incubated with either vehicle, 3 nM or 30 nM netrin-1 for 45 min at 37°C, 5% CO_2_ prior to being placed into the upper chamber of an IncuCyte® ClearView chemotaxis plate (5000 cells/well), which had been coated with Matrigel (50 μg/ml for 30 min at 37°C and 30 min at RT). To assess the uniformity of cell loading in the experiments conducted in Fig 3, the total topside area covered by cells (Total phase object area) at time point zero for each condition and each biological replicate was calculated. Data are mean ± SEM, n = 4 biological replicates with 4 technical replicates per condition.
(EPS)

S3 Fig Netrin-1 has no effect on BMDM viability. (A) BMDMs were seeded into black walled 96 well plates (50,000 cells/well) and left to adhere for 2 hour at 37°C, 5% CO_2_. Cells were then treated with vehicle, 3 nM or 30 nM netrin-1 for 45 min, 4 hours or 24 hours. Cell viability was then determined using the CellTiter-Glo^®^ luminescent cell viability assay. Data are mean SEM, n = 4 biological replicates with 2 technical replicates per condition. Statistical analysis was conducted by two-way ANOVA with Sidak’s multiple comparisons correction. ns P > 0.05.
(EPS)

S4 Fig Netrin-1 has no effect on C5a-induced p38 phosphorylation. (A) BMDMs were seeded into 6 well dishes (2x10^6^ cells/dish) and then treated with either vehicle or 3 nM netrin-1 for 45 min at 37°C, 5% CO_2_. Cells were then stimulated with either vehicle or 10 nM C5a for 5 or 30 min at 37°C, 5% CO_2_. Cell lysates were prepared and western blotting conducted for phosphorylated p38 (phospho-p38) followed by stripping and re-staining for total p38 as a loading control. A blot, representative of n = 2 biological replicates, is shown.
(EPS)

S5 Fig Full uncropped western blots used in this study. (TIF)
