# Supplementary figures and images for: Netrin-1 Reduces Monocyte and Macrophage Chemotaxis towards the Complement Component C5a
Source: PLoS One. 2016 Aug 10;11(8):e0160685. doi: 10.1371/journal.pone.0160685 (PMC4980032; doi:10.1371/journal.pone.0160685)

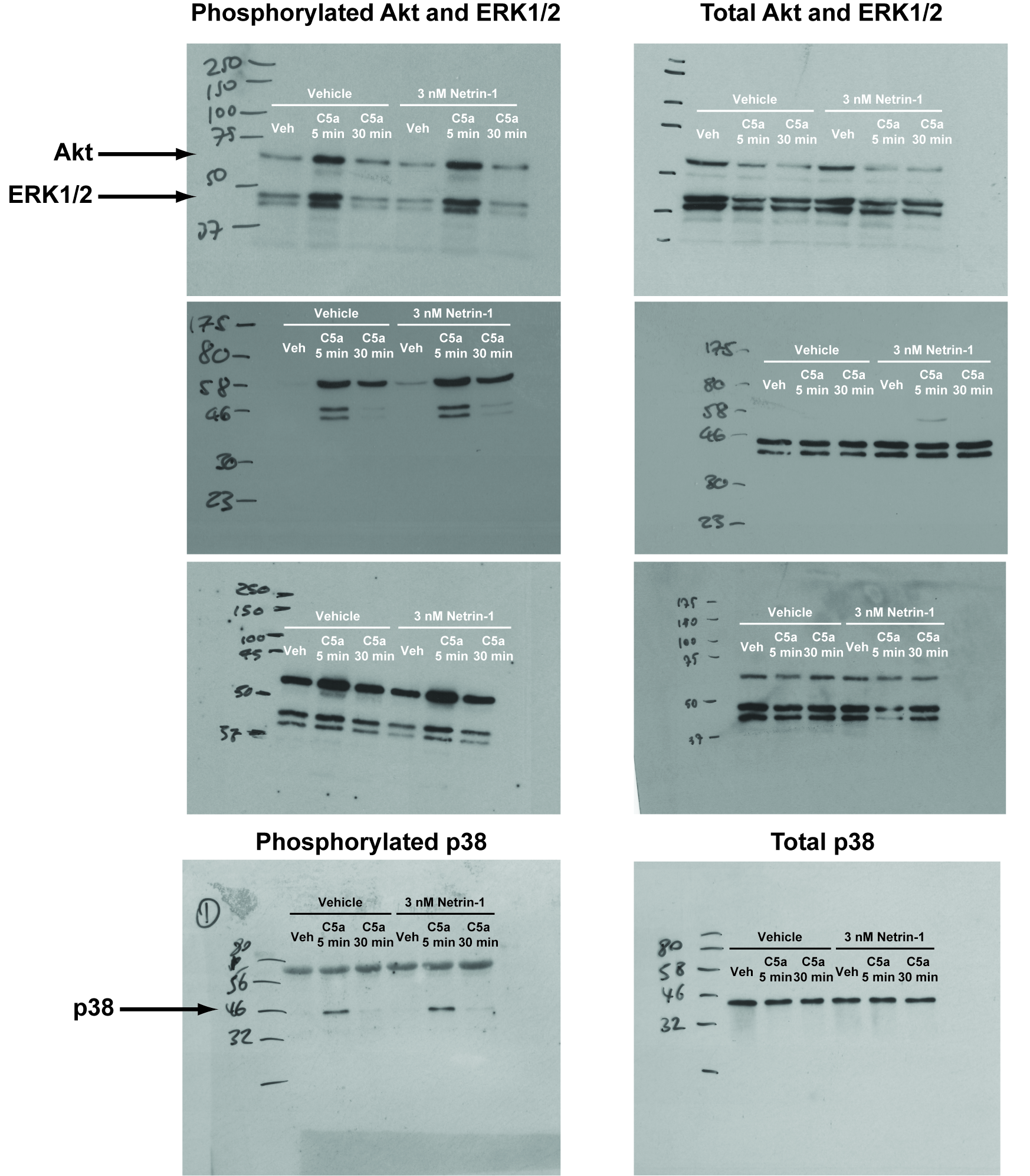

Supplement: S5 Fig — (TIF) [file pone.0160685.s005.tif]
